# Pathogenesis Induced by Influenza Virus Infection: Role of the Early Events of the Infection and the Innate Immune Response

**DOI:** 10.3390/v17050694

**Published:** 2025-05-12

**Authors:** Alicia Helena Márquez-Bandala, Lourdes Gutierrez-Xicotencatl, Fernando Esquivel-Guadarrama

**Affiliations:** 1 Instituto de Biotecnología, Universidad Nacional Autónoma de Mexico, Cuernavaca C.P. 62209, Morelos, Mexico; alicia.marquez@ibt.unam.mx; 2 Laboratorio de Inmunología Viral, Facultad de Medicina, Universidad Autónoma del Estado de Morelos, Cuernava C.P. 62350, Morelos, Mexico; 3 Laboratorio de Virus y Cáncer, Centro de Investigacion Sobre Enfermedades Infecciosas, Instituto Nacional de Salud Publica, Cuernavaca C.P. 62209, Morelos, Mexico; mlxico@insp.mx

**Keywords:** influenza virus, pathogenesis, immune response, cytokine storm, viral load, autoantibodies

## Abstract

Infections by influenza A virus (IAV) are a significant cause of global mortality. The pathogenesis of the infection is usually studied in terms of direct viral-induced damage or the overreactive immune response that continues after the virus is cleared. However, factors such as the initial infectious dose, the early response after infection in different cell types, and the presence of autoantibodies for relevant antiviral cytokines like type I IFNs seem to influence the course of the infection and lead to fatal outcomes. In this article, we address the current knowledge about the early events during influenza virus infection, which are important for their participation in influenza-derived pathogenesis.

## 1. Introduction

IAV is one of the most important human pathogens and a continuous concern to the public healthcare system. Human IAV infections are associated with high morbidity and mortality, especially in children, the elderly, and people with underlying health conditions like heart diseases, diabetes, chronic respiratory conditions, or congenital and acquired immunodeficiencies [1,2,3]. Up until 2020, data on seasonal influenza-associated respiratory deaths allowed the estimation of a global average of 291,243–645,832 cases annually [4]. However, the presence of an important reservoir of IAV in wildlife birds, continuous zoonotic avian influenza infections with H5N1, H5N6, and H7N9 subtypes [5,6], and sporadic zoonotic infections by uncommon subtypes like H10N3 (which display molecular markers associated with mammalian adaptation) [7] raise concern about the possible emergence of an influenza pandemic strain that may result in a very high mortality rate.

IAV infection is associated with a wide spectrum of symptoms, ranging from mild symptoms to severe complications and the development of acute respiratory distress syndrome (ARDS) and respiratory failure. A complex interplay between intrinsic viral pathogenicity, attributable to its tropism for host airway and alveolar epithelial cells, and a dysregulated host innate immune response is known to be implicated in severe and fatal outcomes of IAV infection. However, despite many efforts there still is an incomplete understanding of influenza pathogenesis [8,9]. While many studies have exclusively focused on factors like cytokine storm or overreactive cell responses, early factors that seem determinants of infection establishment are not usually considered. Knowledge of how those early factors contribute to IAV pathogenesis will be useful during both seasonal and influenza pandemics; therefore, in the following sections we will discuss how early factors such as the initial viral dose, infection in different susceptible cells, early cytokine response, chemokine dysregulation, and auto anti-cytokine antibodies may influence IAV infection-derived pathogenesis.

## 2. Influenza A Virus

IAV is a single-stranded negative-sense RNA genome virus belonging to the *Orthomyxoviridae* family. Viral particles possess a host-derived lipid membrane (an envelope) and have eight RNA gene segments (vRNAs) that are known to encode transcripts for at least ten essential viral proteins, and several strain-dependent accessory proteins. Each vRNA is associated with numerous copies of the viral nucleoprotein (NP) and bound to a single copy of the heterotrimeric RNA-dependent RNA polymerase (RdRp) PB1, PB2, and PA, forming the viral ribonucleoprotein complex (vRNP). The viral envelope is supported underneath by the matrix protein 1 (M1), containing the two viral targets of neutralizing antibodies hemagglutinin (HA) and neuraminidase (NA), as well as a few copies of the pH-dependent ion channel protein M2 [10,11].

Upon reaching a potential host cell, the influenza viral cycle is initiated by the interaction between HA and the virus receptor on the cell surface. Human IAV primarily targets cells with sialic acid (SA, N-acetylneuraminicacid) with a 2,6 linkage to underlying sugar chains of glycoproteins in the cell membrane [12]. The HA receptor-binding site attaches the virus to surface glycoconjugates that contain terminal SA residues, triggering virion internalization through receptor-mediated endocytosis. Once inside the cell, endosomal acidification induces an HA conformational change, which exposes the fusion peptide, allowing viral and endosomal membrane fusion. Endosomal low pH also activates the M2 ion channel, and proton influx acidifies the IAV core, allowing the dissociation of vRNP complexes from the M1 protein and their release into the host cytoplasm. The vRNP complexes are imported to the nucleus where genome replication and mRNA synthesis are carried out. The newly synthesized proteins HA, NA, M2, and M1 are transported to the cell membrane, followed by the transport of new vRNA copies that allocate beneath the M1 protein, initiating the viral budding. Finally, the enzymatic activity of viral NA removes sialic acids from both host cells and glycoproteins on the viral membrane, allowing the virion release and preventing its aggregation [11,13].

Early after infection, IAV initiates signaling cascades that trigger the antiviral pro-inflammatory state. Overall, three families of innate Pattern of Recognition Receptors (PRRs), Toll-like receptors (TLRs), RIG-I-like receptors (RLRs), and NOD-like receptors (NLRs), are known to participate during IAV infection. The presence of viral RNA is primarily sensed by TRL3, TLR7 and TLR-8, present in endosomes, which recognize double-stranded RNA and single-stranded RNA, respectively. After viral recognition, TRLs recruit TIR domain-containing adaptor proteins MyD88 or TRIF to initiate signal transduction pathways that ultimately regulate the expression of type I IFN and the production of chemokines and cytokines. Regarding NLRs, retinoic acid-inducible gene I (RIG-I) and melanoma differentiation-associated protein 5 (MDA-5) also participate in the early recognition of IAV RNA species. Transcription of interferon-stimulated genes (ISGs) seems to be the primary role of both RIG-I and MDA-5. In particular, RIG-I signaling is important for the activation of the adaptor mitochondrial antiviral signaling protein (MAVS), which leads to the production of type I IFN, and proinflammatory cytokines through IRF and NFkB, respectively. On the other hand, NLRs, especially NLRP3, form part of the multi-protein inflammasome complexes, and participate in the generation of the active form of IL1-β and IL-18 in a caspase-dependent manner [14,15]. Pyroptosis-mediated cell death has also been linked to activation of the inflammasome complex [16].

## 3. Route of Transmission and the Initial Infectious Dose

As in other viral respiratory infections such as MERS, SARS, RSV, and Rhinovirus [17,18,19], the initial infectious dose, defined as the number of particles that trigger a detectable infection [20], plays a role in the severity of the disease as well as the magnitude of the immune response elicited by IAV infection. In humans, assessing this parameter has been difficult because of the nature of the disease, where the timing and dose of a natural infection cannot be determined accurately. For IAV, human challenge studies have been performed [21,22,23,24,25]. Although these studies have been useful in evaluating viral replication and shedding, as well as providing insights about the immune response to infection, they had usually been performed using a standardized infectious dose of IAV, and the effect of different doses of the same strain was not considered. Thus, mathematical modeling has been useful to overcome some of the limitations of human challenge studies. In this regard, it has been proposed that the increased case fatality rate in susceptible healthy individuals during the 1918 Spanish flu pandemic was associated with infection with higher IAV doses [26]. However, it is to be noticed that in natural infections different factors such as the presence of pre-existing antibodies (generated in response to previous infections), viral strain, and current vaccination status may influence individual response to a high infectious dose [27]. To overcome former limitations, Memoli et al. (2015) [28] performed a healthy volunteer challenge study using different doses of the wild-type A(H1N1)pdm09 IAV strain. The authors showed that typical influenza symptoms (e.g., rhinorrhea, nasal/sinus congestion, sore throat, headache, and fatigue) were more prevalent in individuals infected with higher doses. A stronger immune response, measured as an early rise in inflammatory cytokines, augmented antibody titers against HA and NA viral proteins, and a longer length of viral shedding, was also observed in the previously mentioned group, underlining the impact that individuals initially infected with higher doses represent in disease spread among a natural population.

On the other hand, the mouse model has been useful in extensively examining the effect of initial viral input in inflammatory responses elicited by IAV. In the context of seasonal infection, Marois et al. (2012) [29] demonstrated that the initial infectious dose influences morbidity, lung damage, and lung viral load during IAV infection. Compared with the low-dose infected group (2.52 × 10^2^ PFU), widespread hemorrhagic and inflammatory areas and a higher relative expression of mRNA levels of the inflammatory cytokines TNFα, IL-6, and IL1-β were found in the lungs of mice infected with a high dose of H3N2 X-31 viral strain (2.52 × 10^5^ PFU). Also, although lung viral titers peaked at day 2 post-infection (p.i.) regardless of the initial infectious dose, viral titers in the high-dose infected group reached significantly higher levels. Additionally, the authors evaluated the oxidative stress generated in response to infection (known to participate in oxidative tissue injury during viral infections) and found that iNOS, NQ01, and HO-1 were significantly higher in mice infected with 2.52 × 10^5^ PFU. Recruitment of neutrophils, NK cells, macrophages, and DCs was also higher in this group. Of great relevance, since alternative experiments performed with the PR8 viral strain in both BALB/c and C57BL/6 mice confirmed a similar trend, the effect of a high viral dose in IAV-mediated pathology seems to be independent of genetic background and viral strain. Also, as demonstrated by Moskophidis and Kioussis (1998) [30], an initial high viral dose may also increase the severity of IAV infection by inducing a low CD8^+^ T cell response, which is unable to control viral replication in lung tissue. Using an F5-RAG-1^−/−^ transgenic mouse with a repertoire of peripheral lymphocytes consisting only of cytotoxic lymphocytes (CTLs) that recognize the NP peptide (366–374 aa) of IAV A/NT/60/68, the authors showed that infection with high doses of H3N2 A/NT/60/80 and H3N2 X-31 correlated with more evident viral spread, extensive inflammation and edema, altered lung tissue architecture, and alveoli loss. Since recruited cells display an activation phenotype (upregulation of IL-2R and downregulation of L-selectin), the impaired ability of CTLs to control IAV seems to be related to an overwhelming viral load at the onset of the infection.

It is well known that IAV proteins can contribute to immune evasion, and their role in influenza infection has been well reviewed elsewhere [31]. Therefore, a high initial viral dose may also influence the magnitude of viral antagonism to the host’s innate antiviral response. By using a single-cell approach, Ramos et al. (2019) [32] found that in A549 infected cells the number of viruses infecting a cell (Multiplicity of Infection, MOI) correlates with a higher expression of the NS and PA gene segments, which are known to encode innate immune and host cellular genes antagonist proteins NS1 and PA-X. Furthermore, a negative correlation between NS and PA expression and cellular innate immune gene expression was found in primary normal human bronchial epithelial (NHBE) cells infected with a higher MOI. In particular, the NS segment showed a consistent negative correlation with the transcription of most of the innate immune genes, supporting the hypothesis that viral proteins encoded by this segment might have a role as a negative regulator of innate immune response and cellular gene transcription in natural infections.

**Figure 1:**
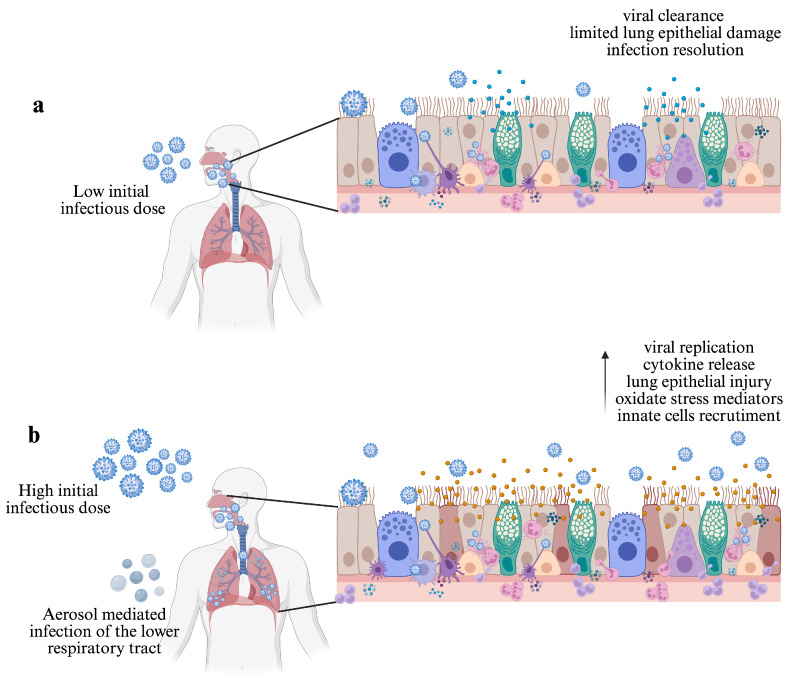
The initial infectious dose and transmission route influences the IAV infection outcome. (**a**) In immune-competent hosts, IAV infection is restricted to the upper respiratory tract. In this context, the innate immune response limits viral replication and lung epithelial damage, leading to infection resolution and host survival. (**b**) High initial infectious doses lead to increased viral replication, impaired viral clearance, augmented immune cell recruitment, epithelial lung damage, and extensive inflammation, compromising host survival. Aerosol-mediated infection of the lower respiratory tract can also lead to an exacerbated immune response even with a relatively minimum dose. Created in https://www.BioRender.com.

Finally, the route of transmission may also have an impact on disease severity and the induction of innate and adaptive immune responses. Through the respiratory route, IAV may be transmitted in a long range of transmission (airborne transmission) by inhalation of an aerosol of non-sedimenting droplets, or in a short range of transmission through direct contact with a droplet spray generated by sneezing, coughing, or talking [33,34]. In this regard, the implications of aerosol transmission have been studied by Smith et al. (2011) [35]. Using a sublethal dose of the mouse-adapted H3N2 X-31 influenza strain, the authors found that, compared with intranasally inoculated mice, the aerosol-inoculated group exhibited more substantial signs of illness and earlier onset of mortality. The authors also found higher levels of the pro-inflammatory cytokine IL-6, more extensive inflammatory cell infiltration, and severe lung pathology. Since aerosol transmission can allow infection of both the upper and lower respiratory tracts of humans [36], it should be considered that even the inoculation of a relatively minimum dose could lead to exacerbated morbidity and pulmonary disease (see Figure 1a,b).

## 4. Effect of the Viral Infection and Replication

### 4.1. Infection on Epithelial Cells

The main target cells for IAV infection and replication are the epithelial cells in the upper respiratory tract (nasal sinuses and the pharynx). In immune-competent hosts, infection with seasonal IAV is usually restricted to this region. However, viral dissemination from nasal epithelia to different lung cavities may occur regarding host respiratory conditions, age, and immune status. Therefore, innate immune responses induced by these cells are critical for both defense against the virus and limiting IAV-induced immunopathology [32,36,37].

**Figure 2:**
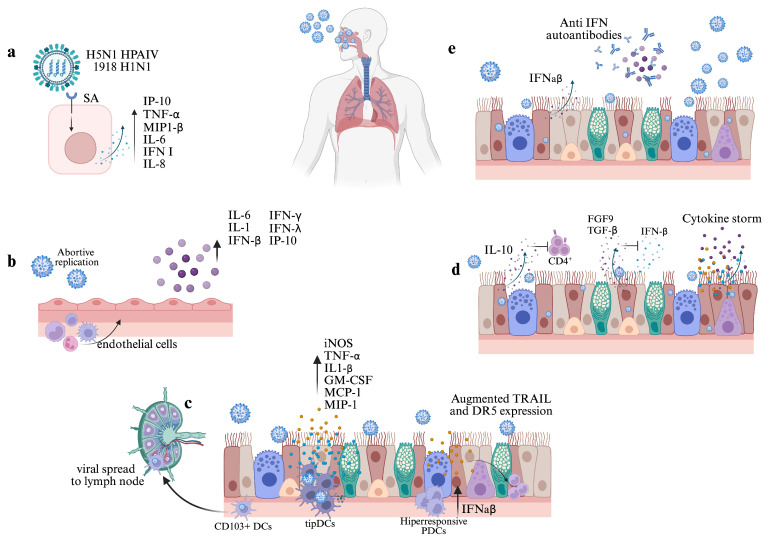
The early events during influenza A virus infection influence immunopathology. (**a**) The interaction between cell receptors and HA from some viral strains promotes the activation of proinflammatory signaling pathways and an augmented immune response. (**b**) Infection of endothelial cells triggers the proinflammatory cytokine response and mediates exacerbated immune cell infiltration. (**c**) During early infection, CD103^+^ DCs migrate to the lymph node and favor viral spread; infection of tipDCs promotes early increased levels of proinflammatory mediators; the presence of hyper-responsive pDCs leads to high levels of IFN-α/-β and increased expression of TRAIL and DR5 in epithelial cells and inflammatory monocytes. (**d**) Dysregulated cytokine response leads to lung tissue inflammation and severe cases; high levels of the anti-inflammatory cytokine IL-10 impair CD4^+^ T cell response; high levels of TGF-β and FGFP contribute to IAV immunopathology by suppressing the early IFN-β response. (**e**) The presence of anti-IFN autoantibodies impairs IFN innate antiviral activity and facilitates IAV replication, leading to critical cases. Created in https://www.BioRender.com.

It is well established that the initial attachment of IAV to the surface and subsequent infection of a susceptible cell is dependent upon interactions between the HA glycoprotein and terminal sialic acid (SIA) residues found on glycoproteins. However, since different IAV strains have different abilities to attach human respiratory tract, a more complex interaction between IAV and glycans has been suggested, where different IAV subtypes could promote activation of specific signaling pathways, ultimately eliciting different immune responses that could lead to immunopathology [12,38,39]. For instance, using recombinant viruses bearing HA from the Highly Pathogenic Avian Influenza Virus (HPAIV) H5N1 A/Vietnam/1203/2004, Ramos et al. (2011) [40] demonstrated that the interaction of IAV with the avian-type receptor (SAα2,3) induced higher levels of IP-10, TNFα, MIP-1β, and IL-6 than H5N1 HA mutant virus with specificity for Saα2,6. Also, the relative expression of former genes and IFN-β, IL-8, and RANTES were augmented more in epithelial respiratory cells infected with H5N1 A/Vietnam/1203/2004 than those infected with H5N1 HA mutant virus. Since these assays were performed at early times post-infection using constructed viruses with a similar backbone, differences between gene expression and pro-inflammatory mediators are independent of viral replication or differences in other viral proteins (see Figure 2a).

Whether the interaction between the HA of other relevant avian strains (H7N9, H9N2, or H10N3) and viral receptors can also induce hypercytokinemia remains unknown. Kobasa et al. (2004) [41] demonstrated that infection of BALB/c mice with reassortant virus bearing HA from the 1918 pandemic virus results in high levels of macrophage-derived chemokines and cytokines, massive infiltration of inflammatory cells, and severe intra-alveolar hemorrhage. Surprisingly, competitive binding assays demonstrated that pandemic HA present in reassortant viruses preferentially recognizes NeuAcα-2,6Gal over NeuAcα-2,3Ga. Therefore, HA from different viral strains seems to play an early important role in influenza immunopathogenesis. Finally, in addition to hyperinflammation, IAV infection alone can lead to the damage and death of epithelial cells, alveolar edema and denudation, pneumocyte hyperplasia, impaired respiratory gas exchange function, and respiratory failure.

### 4.2. Infection in Other Cells of the Respiratory Tract

Other structural cell types found in the respiratory tract like endothelial cells are also involved in early cytokine and chemokine response to IAV infection, innate cell recruitment, and regulation of the cytokine storm. In an in vitro model of primary human lung microvascular endothelial cells (LMECs), Bauer et al. (2023) [42] found that although LMECs are rarely and abortively infected by IAV, high levels of pro-inflammatory cytokines such as IL-6, IL-1, IP-10, IFN-β, IFN-λ, and IFN-γ are produced in response to infection, suggesting that in a natural infection endothelial cells can potentially contribute to systemic inflammation.

Over-recruitment of inflammatory innate cells can also be mediated by endothelial cells, as demonstrated by Teijaro et al. (2011) [43]. Using the C57BL/6J mouse model, the authors found that upon infection with IAV WSN signaling through Sphingosine-1-phosphate (S1P1) mediates macrophage, monocytes, neutrophils, and NK cell infiltration. Moreover, as infection also triggers a pro-inflammatory cytokine response, the administration of S1P1 receptor-specific agonists considerably suppressed cytokine production and cell infiltration, resulting in enhanced mice survival, demonstrating a significant biological role of endothelial cells in early response to infection (see Figure 2b).

Different types of immune cells are also susceptible to influenza infection and may play a role in pathogenesis. For instance, macrophages are among the most studied immune subsets and their relevance during IAV infection has been extensively reviewed [44,45,46]. Several studies performed in vitro using monocyte-derived macrophages have provided information about the potential contribution of these cells to lung pathology. It has been shown that infection upregulates mRNA expression and soluble inflammatory cytokines and chemokines such as type I IFN, TNFα, CCL2, CCL3, RANTES, and IP-10. Moreover, infection with avian strains like H5N1 and H9N2 induces a higher pro-inflammatory response than infection with seasonal IAV strains, which ultimately could account for the severity of avian infections [47,48], although contradictory findings regarding IAV productive infection in different macrophage subsets can be found.

On the other hand, the impact of lung resident macrophages is not usually considered. Alveolar macrophages (AMs) are located on the luminal side of the alveolar niche and function as the first-line defenders of the alveoli and airways. Due to their location, a close relation exists between AMs, capillary endothelial cells, and type I and type II epithelial cells that are relevant to both homeostasis and early response to infections [46,49]. Using C57BL/6 mice, Snelgrove et al. (2008) [50] demonstrated that at steady state lung endothelial cells and type II epithelial cells constitutively express CD200 (also known as OX2), which interact with CD200R on AM surfaces, controlling AM activation and the production of inflammatory cytokines. An absence of CD200R in knockout mice (CD200R^−/−^), or blocking of the receptor with the OX90 antibody, results in an evident weight loss and mortality upon infection with a usually nonlethal dose of influenza X-31 strain. As shown by the authors, morbidity and mortality were related to the amplitude and duration of inflammatory response rather than viral titers, highlighting the relevance of the CD200–CD200R axis for an early control of the inflammatory response during IAV infection. Also, Ettensohn et al. (2016) [51] showed that AM seems to be not permissive to IAV infection. However, an incremented phagocytic activity of AM in response to IAV-infected apoptotic cells was demonstrated by Hashimoto et al. (2007) [52]; this incremented activity may play a role in the inhibition of viral propagation and limiting lung pathology at early stages of the infection.

Finally, dendritic cells (DCs) are considered professional antigen-presenting cells (APCs) and have a crucial role in initiating the adaptive immune response to IAV infection by activating both T and B lymphocytes, but may also serve as direct effector cells in the early innate immune response to infection. DCs are usually classified as conventional dendritic cells (cDCs) and plasmacytoid dendritic cells (pDCs). While cDCs participate in antigen presentation, pDCs display particular functional specializations that enable viral recognition and the production of type I IFN in response to it [53,54].

Regarding cDCs, two major subsets of lung resident DCs, CD103^+^ DCs and CD11b^high^ DC, become infected and migrate to the draining mediastinal lymph nodes after IAV infection. However, Moltedo et al. (2011) [55] demonstrated that in contrast to CD11b^high^ DCs, CD103^+^ mice lung DCs have an attenuated IFNAR signaling response that allows IAV replication. While this characteristic is consistent with the ability of CD103^+^ cells to present antigens to CD8^+^ T cells, the authors highlight that this attribute could be exploited by IAV as a mechanism for viral spread to lymph nodes, leading to infection of other susceptible cells and systemic viral dissemination (see Figure 2c). On the other hand, using C57BL/6J mice Aldridge et al. (2009) [56] showed that during lethal infections with influenza viruses H1N1 A/PuertoRico/8/34 and H5N1 A/Vietnam/1203/2004, a specific subset of TNFα/inducible nitric oxide synthase (iNOS)-producing DCs (tipDCs) accumulate significantly in the lung. This accumulation correlates with early increased levels of pro-inflammatory cytokines and chemokines MCP-1, MIP-1α and -1β, IL-β, IL-6, TNFα, GM-CSF, G-CSF, and KC. A reduced accumulation of tipDCs via suppression of MCP-1 and MCP-3 cytokines in B6 mice results in diminished morbidity and mortality. However, the authors found that tipDCs are relevant for further CD8^+^ CTL activation, highlighting the complex dynamics of cDCs during IAV infection.

As shown by Davidson et al. (2014) [57], the presence of hyper-responsive pDCs could account for increased morbidity and mortality during IAV infection. The authors showed that bone marrow-derived pDCs from mouse strain 129 produce high amounts of IFN-α/-β when exposed to live influenza virus in vitro, and depletion of these cells led to a markedly decreased mice morbidity, significantly reduced levels of inflammatory cytokines in bronchoalveolar lavage (BAL) fluid, and reduced numbers and frequencies of inflammatory monocytes and NK cells. Moreover, an increased expression of TNF-related apoptosis-inducing ligand TRAIL and DR5 receptor was found in inflammatory monocytes and epithelial cells, respectively; the authors showed that this expression is related to the high amounts of IFN-α/-β and directly correlates with staining for cell apoptosis in lung epithelial cells. Therefore, the high amounts of IFN-α/-β produced by pDCs seem to be an upstream deleterious mechanism that ultimately results in high morbidity and mortality mediated by the TRAIL–DR5 interaction. Considering that individuals with high frequencies of pDCs could be more susceptible to severe or fatal outcomes, this observation becomes relevant in the context of influenza infections in the human population.

Lastly, it has been demonstrated that during lethal infections with high doses of influenza virus pDCs can also enhance severity and fatal outcomes by eliminating virus-specific CD8^+^ T cells within the lymph nodes (LNs). Langlois and Legge (2010) [58] found that between days 2 and 4 after infection with a high dose of mouse-adapted H2N2 A/Japan/305/57, a high number of pDCs with augmented expression of FasL can be detected. By performing an ex vivo apoptosis assay, the authors demonstrate that pDCs induced significant levels of FasL–Fas-mediated apoptosis of IAV-specific CD8^+^ T cells after co-culture. When wild-type pDCs were adoptively transferred into a specific mouse lacking functional FasL (gdl mice), the number of CD8^+^ T cells within the LN was significantly reduced, and infection with a high dose of IAV resulted in enhanced mortality. Altogether, these findings suggest that during highly virulent human IAV infections early effector activity of pDCs may contribute to impaired immune response and fatal outcomes (see Figure 2c).

## 5. Early Cytokine Response

Cytokines play a major role in both host defense and immunopathology during IAV infection. Many efforts have been made to characterize the precise cytokine storm mediators involved in human immunopathogenesis; however, a redundancy of signaling pathways, the influence of different virus strains, and impaired immune responses have led to contradictory findings. Nevertheless, it has become clear that excessive early cytokine responses are related to poor medical outcomes [47,58,59,60,61]. For instance, Bermejo-Martin et al. (2009) [62] identified that early Th1 and Th17 hypercytokinemia characterized severe cases of the 2009 H1N1 influenza strain. The authors found that, shortly after the onset of the symptoms, systemic elevation of innate immunity mediators IP-10, MCP-1, and MIP-1β are present in patients with mild and severe symptoms with no significant differences among them. However, in critical and non-critical hospitalized patients high systemic levels of IL-17, TNFα, IL-8, IFN-γ, IL-13, IL-9, and IL-10 can be found, while critical patients showed the highest levels of IL-15, IL-6, and IL-12p70. According to the authors, the dramatic increase in mediators related to Th1 and Th17 in severe patients may reflect a vigorous antiviral host response necessary for viral clearance that ultimately can lead to excessive tissue inflammation.

Similarly, an intensive cytokine induction following the 2009 H1N1 influenza infection was reported by Yu et al. (2011) [63]. Compared with healthy control patients, significantly higher levels of IL-2, IFN-γ, IL-6, TNFα, IL-5, and IL-10 were detected in the serum of patients with mild symptoms. Notably, overwhelmingly high serum levels of IL-6 and IL-10 were observed in patients with severe symptoms, and a positive strong correlation between levels of the formerly mentioned cytokines was identified. Altogether, the authors suggested that increased IL-6 and IL-10 may influence the observed clinical severity in the 2009 H1N1-infected patients.

De Jong et al. (2006) [64] also demonstrated that hypercytokinemia of specific chemokines and cytokines is associated with severe and fatal cases of human H5N1. Levels of IP-10, MIG (CXCL9), IL-18, and MCP-1 measured in peripheral blood were elevated in infected patients, especially in those who died. Also, elevated levels of IL-10, IL-6, and IFN-γ were found. It is to be noted that plasma levels of former cytokines and chemokines correlated with pharyngeal H5N1 viral load. Therefore, the authors proposed that clinical management in these patients should be focused on preventing the intense cytokine response.

It is important to note that in the previously mentioned studies the levels of IL-10, considered an important anti-inflammatory and immunoregulatory cytokine, were elevated in both mild and severe patients. In this regard, according to Sun et al. (2010) [65] a detrimental rather than beneficial role of IL-10 during primary IAV infection can be recognized. Using C57BL/6 IL-10^−/−^ mice, the authors found that the absence of IL-10 results in increased survival, decreased morbidity, and enhanced virus clearance after challenge with the influenza A/PR8/34 strain. According to the authors, the detrimental role of IL-10 was not associated with decreased or altered kinetics of immune cell recruitment to the site of infection. Instead, it seems that IL-10 mediates the suppression of CD4^+^ T cell function, leading to impaired virus-specific antibody production within the lungs and an increased susceptibility to infection. In line with these results, McKinstry et al. (2009) [66] reported increased survival in IL-10-deficient mice infected with high doses of influenza A/Philippines/2/82/x-79 H3N2. The authors found that the absence of IL-10 is not related to differences in the numbers of infiltrating cells or lung pathology but rather leads to a CD4^+^ T-mediated increased expression of Th17-type cytokines that enhances viral clearance and host protection. This supports the hypothesis of an early detrimental role for IL-10 during influenza infection. Since high serum levels of IL-10 can also be found in patients infected with seasonal influenza strains [67], this mechanism could lead to immunopathology during seasonal, pandemic, and avian infections.

Another cytokine usually recognized for its immunoregulatory role during IAV infection is the transforming growth factor β (TGF-β). However, Denney et al. (2017) [68] demonstrated that, by suppressing the early IFN-β response, local epithelial-derived TGF-β (epTGF-β) could act as a pro-viral factor and enhance immunopathology. The authors demonstrated that infection with the X-31 IAV strain (1.2 × 10^4^ TCID50) in C57BL/6 epTGF-β knockout mice (epTGF-βKO) resulted in minimal weight change, less pathological changes within lung tissue, and a reduced viral load. Also, the authors determined that increased *Ifnb1* mRNA expression in epithelial cells and IFN-β levels in bronchoalveolar lavage (BAL) of epTGF-βKO mice can be detected early after infection. Moreover, in epithelial cells significant expression of the mRNA of the interferon-stimulated gene *Oas2* correlates with the increased *Ifnb1* expression. Therefore, protection in mice lacking epithelial TGF-β is mediated by the early antiviral state mediated by IFN-β. In summary, findings regarding the detrimental roles of IL-10 and TGF-β reveal a more complex and, apparently, context-dependent role of anti-inflammatory cytokines during the early phase of IAV infection.

On the other hand, it has been shown that fibroblast growth factor 9 (FGF9) also modulates the early type I IFN response and influences IAV tropism and pathogenesis. By using transgenic FGF9-OE mice, which over-express FGF9 in the conducting airway epithelium, Hiller et al. (2022) [69] found that, in contrast with their control littermates, FGF9-OE mice exhibit increased morbidity, delayed viral clearance, and significantly more histopathology changes related to severe inflammation after infection with the influenza WSN strain. RNA-seq and RT-qPCR analysis of CD45^−^ CD326^+^ CD24^+^ airway epithelial cells revealed that, compared with the control airway epithelial cells, positively enriched pathways in FGF9-OE cells were all related to the innate immune response and IFN signaling. Remarkably, *Infb1*, *Ifnl2*, multiple *Ifna* subtypes, and different antiviral ISGs were upregulated early at day 1 p.i. Taking into account that significantly elevated levels of different cytokines and chemokines, including IL1-β, IL-6, TNFα, IFN-γ, MCP-1, MIP-1α, and MIP-1β were found in lung homogenates of FGF9-OE mice at day 1, the authors concluded that, in response to IAV infection, FGF9 signaling appears to induce an early robust type I IFN response and a detrimental hyperreactive cytokine and chemokine response within the lung epithelium. The authors also demonstrated that in FGF9-OE mice extremely low levels of airway epithelial cells but an increased number of alveolar cells were infected at day 1; therefore, during IAV infection an increased expression of ISGs could protect the airway epithelium, but favors infection of the alveolar cells, resulting in enhanced alveolar inflammation.

Finally, regarding possible mechanisms underlying the cytokine storm, Wan et al. (2021) [70] recently found that the efficient infection of influenza A/Anhui/2013 (H7N9) in C57BL/6 mice triggers Gasdermin E (GSDME)-mediated pyroptosis in alveolar epithelial cells. As a consequence, the release of cytosolic contents induces significantly higher levels of IL1-α, IL1-β, IL-6, IL-10, IL-12, IL-8, G-CSF, KC, MCP-1, and MIP-1α, which ultimately leads to a lethal outcome. According to the authors, the GSDME-mediated pyroptosis may not be induced in infections with other IAV strains. However, strategies designed to prevent GSDME-mediated pyroptosis could be useful in future therapies against H7N9 infections (see Figure 2d).

## 6. Type I IFN and Other Cytokine Autoantibodies

The role of anti-cytokine autoantibodies (ACAAs) in infectious diseases has gained attention in recent years. ACAAs to cytokines like IL-10, GM-CSF, and type I IFN have been detected in healthy individuals and blood donors [71,72]. The presence of this particular group of autoantibodies is usually associated with autoimmune diseases [73,74], and their involvement in several bacterial, fungal, and viral infections has been reviewed elsewhere [75].

ACAAs gained special notoriety during the COVID-19 pandemic when some studies highlighted the relevance of anti-type I IFN antibodies in the outcome of SARS-CoV-2 infection. For instance, Bastard et al. (2020) [76] found that anti-IFN-α and anti-IFN-ω autoantibodies were present in at least 10% of patients with life-threatening COVID-19 and demonstrated that these antibodies had an in vitro neutralizing activity sufficient to impair antiviral activity against SARS-CoV-2. In contrast, auto-IFN antibodies were absent in either asymptomatic or mild COVID-19 patients, showing a correlation between the presence of ACAAs and disease severity. Neutralizing autoantibodies against IL-12p70, IL-22, and IL-6 were also found at a lower level. Moreover, observations made with serum samples obtained from a Chinese cohort also showed that, compared with non-severe COVID-19 patients, a higher proportion of severe/critical patients had elevated titers of IFN-γ autoantibodies, indicating that their presence may be associated with worse clinical manifestations of the disease [77].

In particular, the role of IFN-neutralizing antibodies in influenza infection was first explored in the early 1980s by Hoshino et al. (1983) [78]. By treating CH3/He mice with anti-IFN anti-serum after IAV PR8 infection, the authors found that anti-serum-treated mice have an increased mortality rate, attributable to an impaired ability to contain viral replication. More recently, neutralizing IFN-α and IFN-ω autoantibodies were found in patients with life-threatening influenza pneumonia but no detectable titers of both kinds of autoantibodies or IFN-β autoantibodies were found in individuals with mild influenza infection [79]. Overall, since type I IFN autoantibodies from patients with critical influenza facilitate IAV replication in vitro, it seems that, when present in individuals, autoantibodies impair type I IFN innate antiviral activity, leading to critical cases (see Figure 2d). Regarding ACAAs against other relevant cytokines related to influenza infection, Feng et al. (2023) [80] recently reported the presence of autoantibodies against IFN-α, IFN-γ, SRANK ligand, IL-6, IL-7, IL-12p70, IL-22, and GM-CSF in serum samples of patients infected with influenza virus. Notably, using a cell-based cytokine-blocking assay the authors identified the complete or partial blocking of IL-6 and GM-CSF signaling. However, the functional relevance of these findings remains elusive. Evaluation of larger cohorts will be necessary to determine the prevalence and functional implications of other ACAAs.

## 7. Conclusions

IAV continues to be a threat to public health. Fatal cases are thought to be the result of a complex interaction between host and viral determinants that ultimately lead to pathogenesis. Due to the nature of the disease, some specific aspects of the infection like the initial infectious dose are difficult to determine accurately. However, it becomes evident that predisposing factors like the presence of neutralizing autoantibodies for the major cytokines that participate in the response to infection, different viral strains that preferentially recognize some receptors, and the presence of specific cell subsets like overreactive pDCs could be relevant for early prognosis. Considering knowledge about these early determinants could be useful during both seasonal and pandemic infections.
